# Transpulmonary and pleural pressure in a respiratory system model with an elastic recoiling lung and an expanding chest wall

**DOI:** 10.1186/s40635-016-0103-4

**Published:** 2016-09-20

**Authors:** Per Persson, Stefan Lundin, Ola Stenqvist

**Affiliations:** Department of Anesthesiology and Intensive Care, Sahlgrenska University Hospital, Blå Stråket 5, 413 45 Gothenburg, Sweden

**Keywords:** Lung elastance, Transpulmonary pressure, PEEP step maneuver, Expanding chest wall

## Abstract

**Background:**

We have shown in acute lung injury patients that lung elastance can be determined by a positive end-expiratory pressure (PEEP) step procedure and proposed that this is explained by the spring-out force of the rib cage off-loading the chest wall from the lung at end-expiration. The aim of this study was to investigate the effect of the expanding chest wall on pleural pressure during PEEP inflation by building a model with an elastic recoiling lung and an expanding chest wall complex.

**Methods:**

Test lungs with a compliance of 19, 38, or 57 ml/cmH_2_O were placed in a box connected to a plastic container, 3/4 filled with water, connected to a water sack of 10 l, representing the abdomen. The space above the water surface and in the lung box constituted the pleural space. The contra-directional forces of the recoiling lung and the expanding chest wall were obtained by evacuating the pleural space to a negative pressure of 5 cmH_2_O. Chest wall elastance was increased by strapping the plastic container. Pressure was measured in the airway and pleura. Changes in end-expiratory lung volume (ΔEELV), during PEEP steps of 4, 8, and 12 cmH_2_O, were determined in the isolated lung, where airway equals transpulmonary pressure and in the complete model as the cumulative inspiratory-expiratory tidal volume difference. Transpulmonary pressure was calculated as airway minus pleural pressure.

**Results:**

Lung pressure/volume curves of an isolated lung coincided with lung P/V curves in the complete model irrespective of chest wall stiffness. ΔEELV was equal to the size of the PEEP step divided by lung elastance (EL), ΔEELV = ΔPEEP/EL. The end-expiratory “pleural” pressure did not increase after PEEP inflation, and consequently, transpulmonary pressure increased as much as PEEP was increased.

**Conclusions:**

The rib cage spring-out force causes off-loading of the chest wall from the lung and maintains a negative end-expiratory “pleural” pressure after PEEP inflation. The behavior of the respiratory system model confirms that lung elastance can be determined by a simple PEEP step without using esophageal pressure measurements.

## Background

Transpulmonary driving pressure is a key factor in ventilator-induced lung injury and also for rational setting of positive end-expiratory pressure (PEEP) [1–3]. The transpulmonary pressure is traditionally calculated as the product of end-inspiratory airway pressure and the ratio of lung to respiratory system elastance [4–6]. The ratio of lung and respiratory system elastance is in turn estimated from the change in airway and esophageal pressure during tidal volume inflation. Unfortunately, due to difficulties in positioning of the catheter as well interpretation of data, esophageal pressure measurements at the bedside is still rarely performed [7].

We have recently shown that it is possible to determine lung elastance without using esophageal pressure measurements using a simple PEEP step procedure [8, 9]. We have proposed that this phenomenon is explained by the spring-out force of the rib cage off-loading the chest wall from the lung at end-expiration. The consequence is that the end-expiratory pleural pressure does not change when end-expiratory lung volume is increased by raising PEEP. Consequently, end-expiratory transpulmonary pressure increases as much as PEEP is increased. This has previously been described by Rahn and coworkers 1946, where the chest wall pressure/volume curve was determined during end-expiratory relaxation at different lung volumes [10] without using esophageal or pleural pressure measurements. The fundamental effects of the expansive chest wall on lung mechanics have been discussed [11], but skepticism concerning the clinical effects seems to prevail.

The aim of the present study was to examine the hypothesis that the expansive chest wall is the cause of the unchanged pleural pressure after PEEP inflation in contrast to the changes in pleural pressure during tidal inflation. To study this, a *respiratory system* model based on Newtonian laws of mechanics [12], with an elastic recoiling lung, an expanding chest wall, and a “pleural” space in between, was designed and studied during tidal and PEEP inflation.

## Methods

### The respiratory system model

**Fig. 1:**
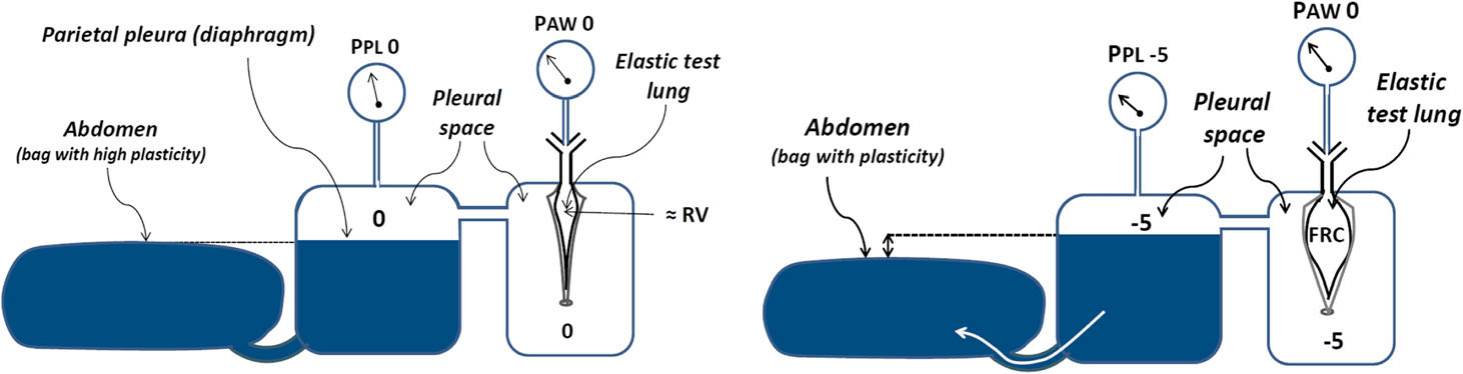
Respiratory system model. *Left panel*: model with no expanding force of the chest wall, where the lung volume is at residual volume and pleural pressure is zero. *Right panel*: model with evacuated pleural space, with a pressure of −5 cmH_2_O, which causes a higher fluid level in the plastic container than in the abdominal sack. As a consequence, the fluid in the plastic container wants to flow towards the sack, imitating the rib cage spring-out force, while the lung is inflated by a positive transpulmonary pressure of 5 cmH_2_O (zero airway pressure minus −5 cmH_2_O pleural pressure), to functional residual capacity (FRC). Nominal lung compliance of 19, 38, and 57 ml/cmH_2_O was achieved by using one, two, or three test lungs in parallel. A stiff chest wall was achieved by strapping hard board on the outside of the pleural space

A model including the contra-directional forces of the recoiling lung and the spring-out force of the rib cage and the abdomen, which can be regarded as a mainly fluid filled container being attached to the caudal rim of the rib cage [10, 13, 14] was designed (see Fig. 1). In this model, the spring-out force of the rib cage created a negative pressure in the pleural space. The abdominal content is displaced caudally during inflation, rather than being inflated like the lung [15]. Test lungs (Maquet CC, Solna, Sweden) with a nominal compliance of 19 ml/cmH_2_O were used as the elastic recoiling lung in the model. The elastic recoil of the test lungs used is not achieved by using an elastic balloon but rather by having a fairly non-elastic balloon placed between two elastic Teflon plates.

One, two, or three test lungs were connected in parallel obtaining a nominal lung compliance of 19, 38, or 57 ml/cmH_2_O. The test lungs were placed in an airtight box, which was connected with a 22 mm diameter tubing to the upper part of a semi-stiff plastic container, which was three quarter filled with water.

In a patient less than half of a volume increase, tidal or PEEP inflation is directed towards the diaphragmatic area and thereby affecting the abdomen. The rest of the volume increase occurs towards the chest wall. The displacement of the diaphragm in caudal direction causes a slight rise of the upper surface of the abdomen. This rise is dependent on the size of the upper surface area of the abdomen. In the model, all volume increase is directed towards the “diaphragm” and the abdominal container as it was not possible to build a separate chest wall and diaphragm. To compensate for this, the diaphragmatic surface area was made 1500 cm^2^, twice the size in an adult person. A water-tight sack with good plasticity and a volume of 10 l, equivalent to the volume of the abdominal cavity and an upper surface area of 1800 cm^2^, double the upper surface of the abdomen of an adult person [16, 17], was connected to the bottom of the plastic container by a 22-mm diameter tubing with a valve with adjustable resistance. The space above the water surface in the plastic container, the tubing, and the box in which the test lungs were placed constituted the pleural space.

Chest wall elastance could be increased by strapping hard board on the outside of the pleural space, i.e., the plastic container and the lung box.

The contra-directional forces of the recoiling lung and the expanding chest wall were obtained by evacuating the pleural space through suctioning to a pressure of −5 cmH_2_O, which caused a higher water level in the plastic container than in the abdominal sack. As a result of the negative pleural pressure, the lung was “inflated” to functional residual capacity (FRC), where the end-expiratory transpulmonary pressure is equal to the end-expiratory airway pressure minus the pleural pressure, 0 − −5 = 5 cmH_2_O (Fig. 1).

### Measurements

Airway pressure was measured by the Flow-I anesthesia machine (Maquet Critical Care, Solna, Sweden) and presented by the Maquet software. End-expiratory pressure was used for calculations of lung elastance and transpulmonary pressures. Pleural pressure was measured in the pleural space by a standard pressure receptor (PVB Medizintechnik, Kirchseeon, Germany) and monitor (S/5, GE Healthcare, Helsinki, Finland). For volume measurements, the pneumotachograph of the ventilator of the Flow-I anesthesia machine (Maquet Critical Care, Solna, Sweden) was used. The change in end-expiratory lung volume (ΔEELV) following an increase/decrease in PEEP was determined as the cumulative difference in inspiratory and expiratory volume during the first 15 breaths following a PEEP change using a dedicated software provided by Maquet CC [18]. The software collects data from the Servo-I or the Flow-I about pressures and flow at a frequency of 100 Hz. The software analyzes the pressure signal and provides the actual measured level of PEEP and senses for changes. If there is a change of PEEP any contribution to the ΔEELV are summed up during 15 breaths. The inspiratory (VTi) and expiratory (VTe) tidal volumes are calculated by the summation of flow samples from the inspiratory and expiratory phase, respectively. The contribution to the ΔEELV (VTi-VTe) after a change of PEEP are compensated for any offset, and the eventual offset are decided during the five breaths following the 15 included in the summation at the new PEEP level.

### Calculations

Tidal total respiratory system, chest wall, and lung elastance were calculated as tidal airway pressure variation, tidal pleural pressure variation, and the difference between tidal airway and pleural pressure variation divided by tidal volume (ΔPAW/VT, ΔPPL/VT, (ΔPAW − ΔPPL)/VT), respectively.

Lung elastance was also calculated as the change in end-expiratory airway pressure divided by the change in end-expiratory lung volume (ΔPEEP/ΔEELV) induced by an increase or a decrease in PEEP.

### Experimental procedure

The respiratory system model was ventilated by a Flow-I anesthesia machine (Maquet CC, Solna, Sweden) in volume control ventilation with a tidal volume of 300 ml, respiratory rate of 15/min, I:E ratio of 1:2, and an end-inspiratory pause of 10 % of the breathing cycle.

### Isolated lung

FRC was determined in isolated lung by measuring change in lung volume (ΔEELV) induced by a PEEP step from zero to 5 cmH_2_O. Lung compliance was determined by ventilation of 1, 2, and 3 test lungs disconnected from the plastic container and “abdominal” sack, i.e., with isolated lung, where airway pressure equals transpulmonary pressure. As the transpulmonary pressure at FRC was ≈5 cmH_2_O, PEEP steps 5-9-5, 5-13-5, and 5-17-5 cmH_2_O were performed in the isolated lung, corresponding to 0-4-0, 0-8-0, and 0-12-0 cmH_2_O with connected pleural space and abdomen. The PEEP steps were performed with one, two, and three test lungs connected, except when one test lung was used, where the 5-17-5 cmH_2_O steps were omitted as 17 cmH_2_O in PEEP resulted in end-inspiratory volume above the capacity of a single test lung.

### Lung(s) together with a normal or stiff chest wall

PEEP steps 0-4-0, 0-8-0, and 0-12-0 cmH_2_O were performed with one, two, or three test lungs connected. When one test lung was used, the 0-12-0 cmH_2_O step was omitted as 12 cmH_2_O in PEEP resulted in end-inspiratory volume above the capacity of a single test lung. All PEEP step sequences were performed with “normal” chest wall and with stiff chest wall.

### Data analysis and statistics

Each PEEP step procedures were run three times, and the average of these runs was used as the result for a PEEP step. As the “pleural” pressure might not be exactly the same after changing between one, two, and three lungs and stiff and normal chest wall, SD is reported as the variation in results when mean results are reported from the totally six procedures: one lung stiff chest wall (CW), one lung normal CW, two lungs stiff CW, two lungs normal chest wall, three lungs stiff CW, and three lungs normal chest wall. Correlation between measured ΔEELV using spirometry and ΔEELV calculated as change in PEEP divided by lung elastance (determined as the difference in airway and pleural driving pressure divided by tidal volume) was analyzed using linear regression analysis.

## Results

**Fig. 2:**
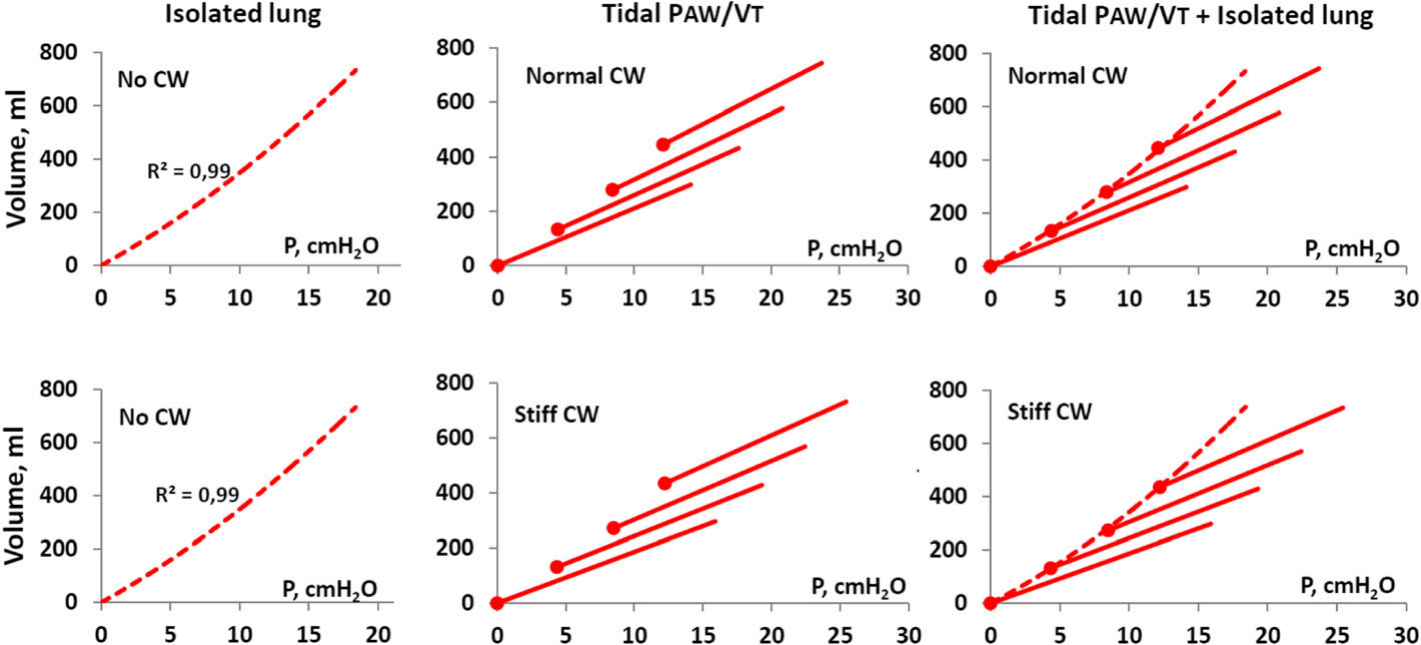
*Left panels*: shows airway pressure/volume (P/V) curve in an isolated lung (no CW), with nominal compliance of 38 ml/cmH_2_O (two test lungs), where the airway pressure equals the transpulmonary pressure. *Mid panels*: tidal airway (total respiratory system) P/V curves at 0, 4, 8, and 12 cmH_2_O of end-expiratory airway pressure (PEEP; *filled circles*). *Upper mid panel*: normal chest wall. *Lower mid panel*: stiff chest wall. *Right panels*: tidal airway (respiratory system) P/V curves with superimposed isolated lung P/V curve (dashed line) from end-expiration at ZEEP to end-inspiration at PEEP 12 cmH_2_O. *Upper right panel*: normal chest wall. *Lower right panel*: stiff chest wall. Note that irrespective of whether the chest wall is normal or stiff, the isolated lung P/V curve is aligned along the tidal end-expiratory airway P/V points

In figures, all curves are starting from FRC but for clarity depicted as starting from zero volume. Pressure/volume curves from ventilation of an isolated “lung,” a lung with intact chest wall ventilated at different PEEP levels as well as lungs with a stiff chest wall are shown in Fig. 2. Note that the isolated lung P/V curve without chest wall, where airway pressure is equal to transpulmonary pressure, coincides with the end-expiratory airway P/V points irrespective of the stiffness of chest wall. The airway pressure/volume curves at increasing PEEP levels were seemingly successively left-shifted irrespective of normal or stiff chest wall in the same way as seen in patients [19–25] (Fig. 2, mid panels). The end-inspiratory airway P/V points are right shifted from the lung (isolated) P/V curve as a consequence of the influence of the chest wall [26] (Fig. 2).

**Fig. 3:**
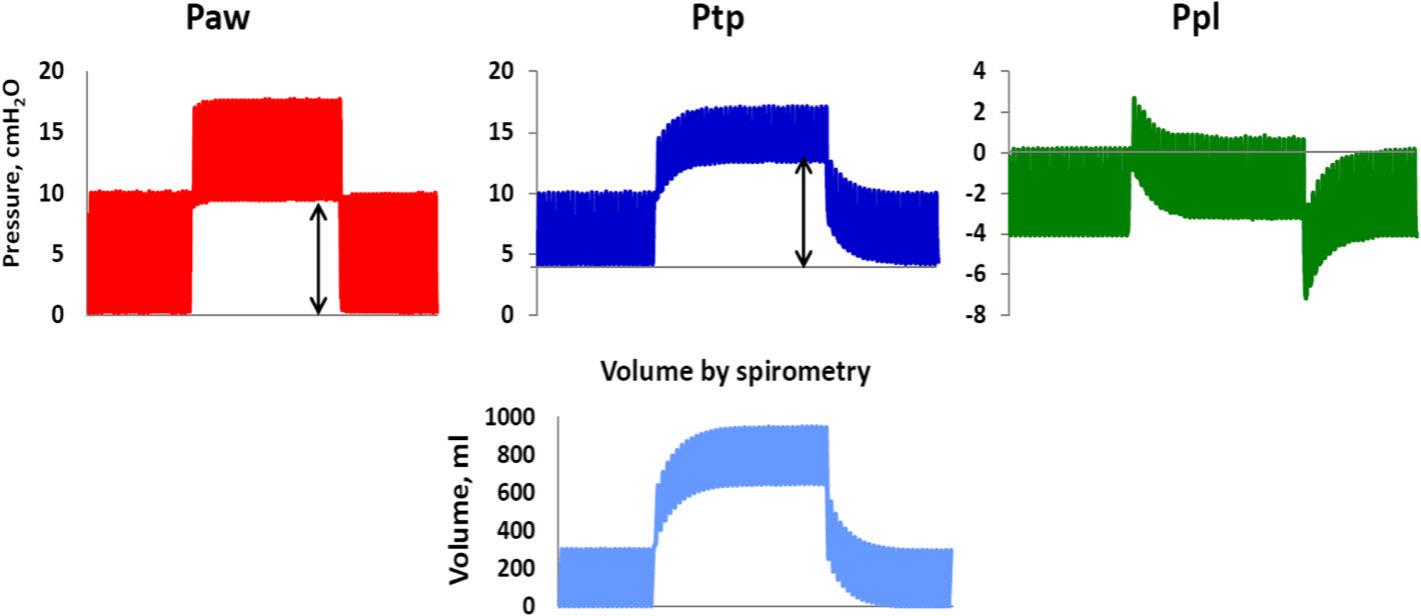
Shows airway pressure and lung volume changes before, during, and after a PEEP step up and down in the model with one test lung and normal chest wall. *PAW* airway pressure (*red*), *PTP* transpulmonary pressure (*dark blue*), *PPL* pleural pressure (*green*). Volume change (*light blue*). *Black arrows* shows the change in PEEP and PTP, which indicate that end-expiratory PTP changes as much as end-expiratory airway pressure (PEEP) is changed

Figure 3 shows the changes in airway pressure, transpulmonary pressure, and pleural pressure and lung volume in the lung model. After increasing PEEP, there was an initial increase in end-expiratory pleural pressure. From this, still negative end-expiratory pleural pressure level, the pressure subsided back towards the baseline negative value of −5 cmH_2_O. Simultaneously, the end-expiratory lung volume measured with spirometry continued to increase with a concomitant increase in end-expiratory transpulmonary pressure (Fig. 3).

The increase in end-expiratory transpulmonary pressure when increasing PEEP from zero end-expiratory pressure (ZEEP) to 4.4 ± 0.1, 8.4 ± 0.2, and 12.2 ± 0.1 cmH_2_O was 4.2 ± 0.1, 8.0 ± 0.2, and 11.4 ± 0.2 cmH_2_O, respectively with normal and stiff chest wall when the model was used with one, two, or three test lungs in parallel with a nominal lung compliance of 19, 38, and 57 ml/cmH_2_O. A step change of PEEP of 0-4-8-12 cmH_2_O, where each step was 4.1 ± 0.3 cmH_2_O, resulted in a step change in transpulmonary pressure of 3.9 ± 0.3 cmH_2_O. The corresponding increase in end-expiratory pleural pressure was 0.2 ± 0.1 cmH_2_O.

The change in end-expiratory lung volume (ΔEELV) of all PEEP steps with one, two, and three lungs and normal and stiff chest wall measured with the ventilator pneumotachograph and ΔEELV calculated as the change in PEEP divided by the lung elastance, ΔPEEP/EL, where lung elastance was determined as the difference in airway and pleural driving pressure divided by the tidal volume (ΔPAW – ΔPPL)/VT, were closely correlated, *Y* = 1.05*X*, *r*^2^ = 0.99.

**Fig. 4:**
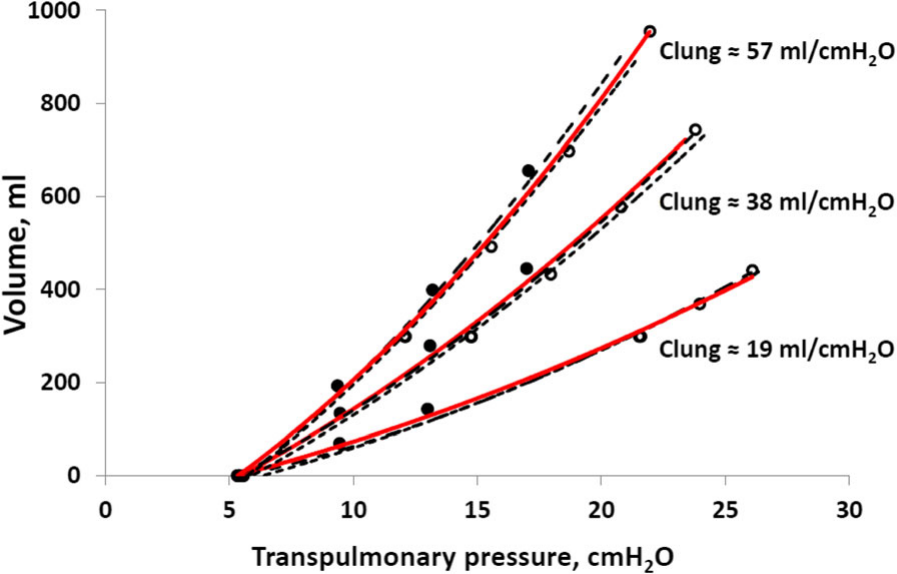
Best fit lung P/V curves from PEEP steps of 4, 8, and 12 cmH_2_O in isolated lung (*red*), normal chest wall stiffness (*long dash*, *black*), and extra stiff chest wall (*short dash*, *black*). *Filled circles* indicate end-expiratory P/V points and *open circles* end-inspiratory P/V points of isolated lung. Note that end-expiratory and end-inspiratory transpulmonary P/V points are aligned on a common transpulmonary P/V curve, and consequently, the transpulmonary pressure at a certain lung volume is independent of whether this specific lung volume is a result of a tidal inflation or PEEP inflation

In the isolated lung without a chest wall, a baseline PEEP of 5 cmH_2_O was applied to achieve a volume corresponding to lung volume (FRC) at a pleural pressure of −5 cmH_2_O in the complete model with lung pleura and abdominal compartment. A PEEP of 5 cmH_2_O in the isolated lung resulted in a FRC of 92, 167, and 242 ml with one, two, or three test lungs with a nominal lung compliance of 19, 38, and 57 ml/cmH_2_O respectively. Lung P/V curves obtained during ventilation of an isolated lung with one, two, or three test lungs were similar to the corresponding lung P/V curves when the chest wall complex was connected, irrespective of if the chest wall was normal or stiff (Fig. 4).

**Fig. 5:**
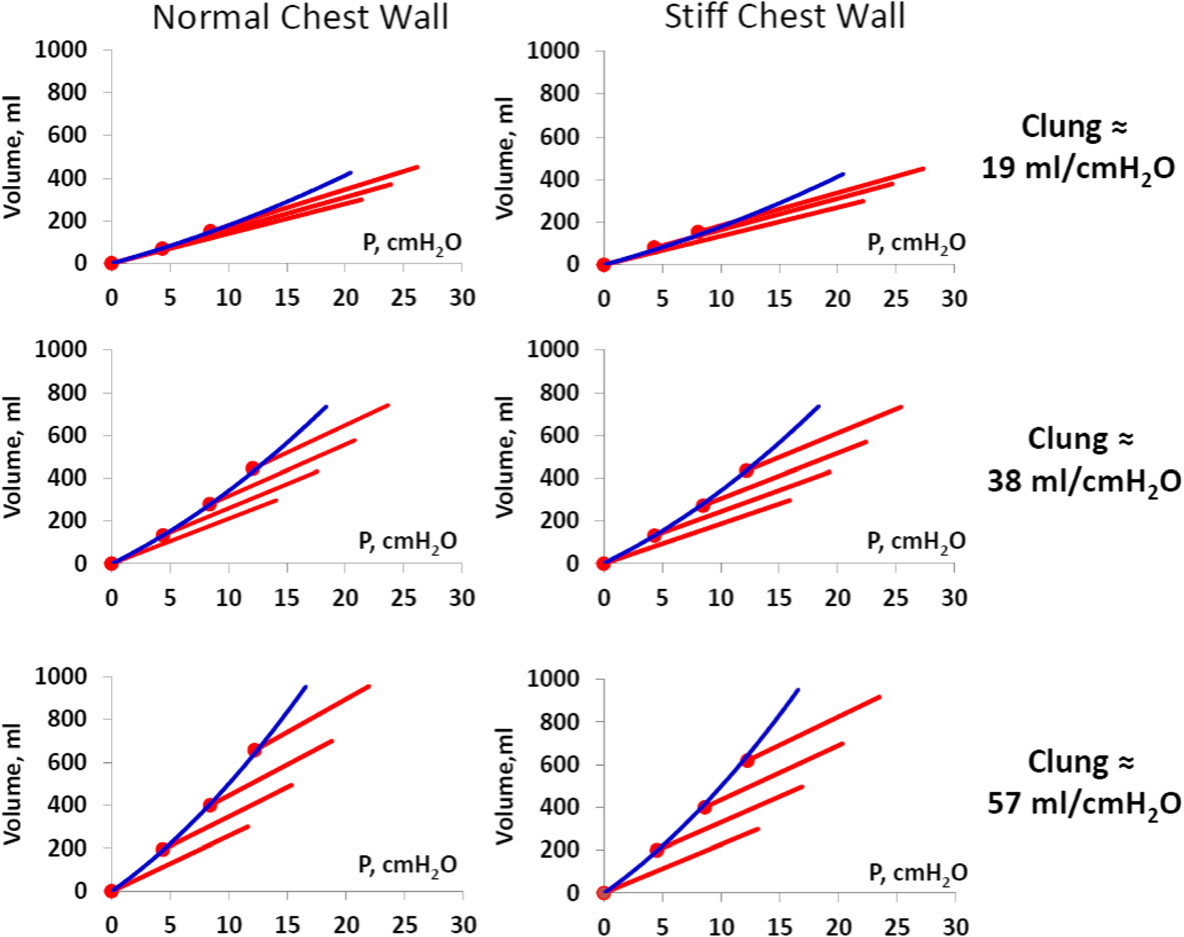
Tidal airway P/V curves (*red*) starting from PEEP levels of 0, 4, 8, and 12 cmH_2_O and lung P/V curves (*blue*) starting from from ZEEP to end-inspiration at the highest PEEP level. *Filled circles* = end-expiratory airway P/V points. Note that the lung P/V curves have been transpositioned in parallel from their actual starting point at ≈5 cmH_2_O to zero to visualize that the slope of the lung P/V curve is identical to the slope of the end-expiratory airway P/V points (for actual position of lung P/V curves, see Fig. 7). Note that the lung P/V curve during PEEP inflation is unaffected by stiffness of the chest wall

**Fig. 6:**
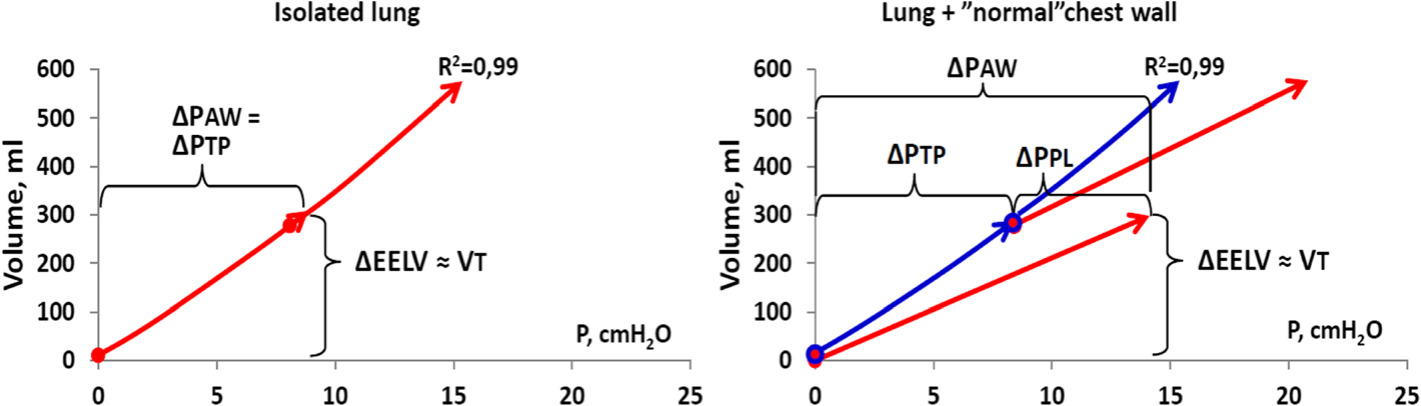
*Left panel*: isolated lung, *right panel*: lung and chest wall. Tidal airway P/V curves (*red arrows*) and tidal lung (transpulmonary) P/V curves (*blue arrows*). In the isolated lung without a chest wall, the airway P/V curve is equal to the transpulmonary P/V curve. The tidal volume from the low PEEP was almost equal to the end-expiratory lung volume change between the ZEEP and a PEEP of 8 cmH_2_O when the chest wall stiffness was normal and lung compliance was 38 ml/cmH_2_O. The end-inspiratory transpulmonary pressure of the tidal volume from ZEEP is close to the end-expiratory transpulmonary pressure at PEEP of 8 cmH_2_O. The lung P/V curves of the isolated lungs (*red arrows*, *left panel*) are identical to the lung P/V curves (*blue arrows*, *right panel*) of the complete model (lung, chest wall and abdomen) in the *right panel*. The end-inspiratory airway pressure of a tidal volume from ZEEP is right shifted from the end-expiratory airway P/V point of PEEP 8 cmH_2_O due to the influence of the chest wall with a pressure equal to the change in pleural pressure (ΔPPL). Note that the lung P/V curves have been transpositioned in parallel from its actual starting point at ≈5 cmH_2_O to zero in order to visualize that the slope of the lung P/V curve is identical to the slope of the end-expiratory airway P/V points (for the actual position of lung P/V curves, see Fig. 7)

Tidal airway P/V curves at increasing PEEP levels are seemingly successively left-shifted, but in reality, it is the end-inspiratory airway P/V points that are right shifted from the lung P/V curve (Figs. 5 and 6).

**Fig. 7:**
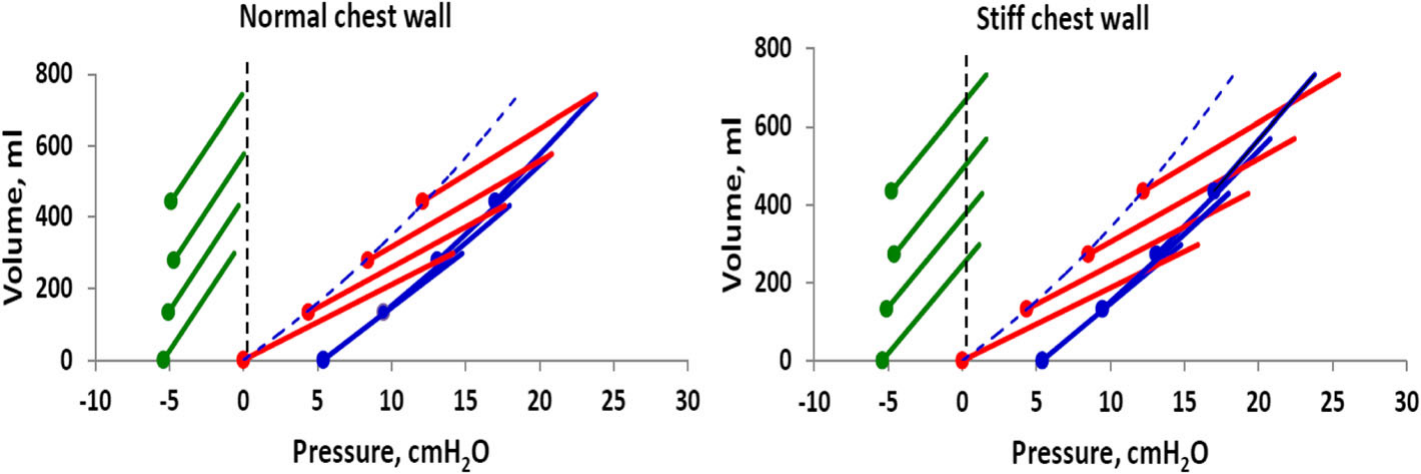
Tidal airway P/V curves (*red*), chest wall P/V curves (*green*), and lung P/V curves (*blue*) in model with normal (*left panel*) and stiff chest wall (*right panel*). End-expiratory P/V points are marked with *filled circles. Blue dashed line*: best fit lung P/V curve transpositioned to start at zero pressure. Note that the best fit lung P/V curve is passing through the end-expiratory airway P/V points and that the end-expiratory pleural pressure remain unchanged and negative when increasing PEEP, indicating an negligible end-expiratory chest wall elastance

Tidal chest wall elastance of the model was 18 cmH_2_O/L with normal chest wall stiffness and 24 cmH_2_O/L with increased chest wall stiffness, irrespective of PEEP level (Fig. 7).

End-expiratory chest wall elastance, between 0 and 12 cmH_2_O in PEEP, calculated as the change in end-expiratory pleural pressure divided by the change in end-expiratory lung volume, ΔPPLEE/ΔEELV, was 1.5 cmH_2_O/L, corresponding to an end-expiratory chest wall compliance of 637 ± 72 ml/cmH_2_O, irrespective of tidal chest wall elastance.

## Discussion

We have constructed a model of the respiratory system with a recoiling lung and an expanding chest wall, where tidal airway (total respiratory system) pressure/volume curves are seemingly left-shifted when increasing PEEP in accordance with what is seen in patients [21, 22, 24, 25, 27]. A change in end-expiratory airway pressure, PEEP with an ensuing inflation of the lung, causes a negligible increase in end-expiratory “pleural” pressure, indicating that end-expiratory elastance of the chest wall is close to zero, i.e., the end-expiratory chest wall compliance is extremely high. As a consequence, the end-expiratory transpulmonary pressure increases as much as PEEP is increased. The increase in end-expiratory lung volume following a PEEP step is determined by the size of the change in PEEP and *lung* elastance. If the change in volume is measured, lung elastance can be determined as the change in PEEP divided by the change in end-expiratory lung volume, ΔPEEP/ΔEELV. Thus, we do not need to know pleural or esophageal pressure to estimate lung elastance and changes in transpulmonary pressure.

### Tidal and end-expiratory chest wall elastance

The expanding chest wall has a fundamental physiological role in counteracting the recoil of the lung at end-expiration, i.e., functional residual capacity (FRC). This has previously been described in a number of classical studies and in textbooks of physiology [10, 13, 28–31] but have only recently been introduced in studies of mechanical ventilation [8, 9, 11].

The outward directed force of the chest wall has a pulling effect on the lung, which in itself strives to recoil to a lower volume, the minimal volume, slightly below the residual volume. The net effect of these contra-directional forces is that a negative pleural pressure and an equally large positive transpulmonary pressure are created, 5–10 cmH_2_O [29–32]. Thus, instead of compressing, squeezing, or leaning at the lung at end-expiration, the chest wall tends to expand the lung at end-expiration as long as the end-expiratory lung volume, irrespective of the reason for the increased volume, emphysema or PEEP inflation, is below the resting volume of the chest wall at 70–90 % of total lung capacity (TLC) [10, 30, 31, 33–36]. The resilience of the chest wall originates in the ribs, ligaments, and cartilages of the rib cage, which are unaffected in severe acute lung injury (ARDS) [11]. This means that the resting volume of the chest wall, i.e., the volume where the chest wall does not strive outwards any more, is around 3 l above FRC, also in respiratory failure patients. As the mean pleural pressure at FRC is around −5 and 0 cmH_2_O at the chest wall resting volume, the end-expiratory chest wall compliance is 3000/5 ≈ 600 ml/cmH_2_O estimated from the chest wall relaxation P/V curve as described by Rahn and coworkers in 1946 [10]. In emphysema patients, who were subjected to lung reduction surgery, end-expiratory chest wall compliance is estimated to be 700 ml/cmH_2_O [37]. In the present lung/chest wall/abdomen model, the end-expiratory chest wall compliance was also very high, i.e., around 600 ml/cmH_2_O, confirming that the geometry of the model reflects chest wall mechanics in humans. Between FRC and the resting volume of the chest wall pleural pressure is negative, and the chest wall strives outwards at end-expiration, irrespective of the pressure inside the lung, of which there are no receptors in the chest wall [10, 31, 37, 38]. This indicates that the chest wall has a negligible influence on the lung during end-expiration as a result of the rib cage spring-out force.

Tidal pleural pressure variations, the chest wall-driving pressure, reflect the force needed to displace a weight, the chest wall, and the abdominal content during *inspiration*, rather than inflating a recoiling structure. As the weight of the chest wall and the abdomen does not change with changing PEEP, tidal chest wall elastance is almost constant during increasing PEEP levels in the respiratory system model, as also reported for patients in several studies [8, 9, 25, 39, 40] (Fig. 7). During an inspiration from ZEEP/FRC pressure/volume equilibrium, pleural pressure increases from a negative pressure level to a less negative pressure level, or if the chest wall is very stiff, to a positive end-inspiratory pleural pressure (Figs. 3 and 7). But, during the expiration, the lung will recoil back to the baseline, and from a volume point of view, the interior volume of the chest wall is always essentially equal to the lung exterior volume; the lung will pull the chest wall back to baseline volume. In doing so, pleural pressure will return to a *negative* level because of the rib cage spring-out force opposing the recoil of the lung.

When PEEP is stepwise increased, the ventilator expiratory valve is closing at the selected, new PEEP level during the first expiration after increasing PEEP, and a volume corresponding to total respiratory system compliance times the change in PEEP is retained in the lung (Fig. 3). As long as this first expiration lung volume increase does not cause the end-expiratory lung volume to exceed the chest wall resting volume at 70–80 % of TLC, the end-expiratory pleural pressure will be negative when the expiratory valve closes (Figs. 3 and 7), indicating that the rib cage spring-out force is active. During the ensuing breaths, the end-expiratory lung volume increases breath by breath and the end-expiratory pleural pressure subsides back to or close to the baseline negative pleural pressure level and a new P/V equilibrium is reached. PEEP inflation of the lung is a pressure control inflation of the lung with constant airway pressure, and end-expiratory airway pressure is the sum of end-expiratory transpulmonary and end-expiratory pleural pressure. As end-expiratory transpulmonary pressure increases breath by breath, when the end-expiratory lung volume increases breath by breath, end-expiratory pleural pressure must decrease after the initial first expiration increase, as much as the transpulmonary pressure increases (Fig. 3). If the chest wall behaved as an elastic entity recoiling to a lower volume like the lung, end-expiratory chest wall elastance, ΔPPL_EE_/ΔEELV, would be equal to tidal chest wall elastance, ΔPPL/VT, like end-expiratory lung elastance, ΔPTP_EE_/ΔEELV, is equal to tidal lung elastance ΔPTP/VT. Consequently, total end-expiratory elastance would be the sum of tidal chest wall and tidal lung elastance, total respiratory system elastance (ETOT) = ΔPPL/VT + ΔPTP/VT and the change in end-expiratory lung volume change following a PEEP increase could not exceed the change in PEEP (=ΔPAW_EE_) divided by the total respiratory system elastance, ΔPEEP/ETOT. However, such a low change in end-expiratory lung volume is only present in isolated lung, where total respiratory system elastance is the same as lung elastance, as seen when ventilating isolated test lungs in the model (Fig. 2). In patients, the change in end-expiratory lung volume is always larger than ΔPEEP/ETOT, except in isolated lung, as reported in multiple studies [20, 21, 22, 27] and determined by the size of the PEEP step and lung elastance, ΔEELV = P ΔPEEP/EL, as seen in the model and in accordance with findings in previous studies in pigs and acute lung injury (ALI) patients [8, 9].

### Transpulmonary pressure

**Fig. 8:**
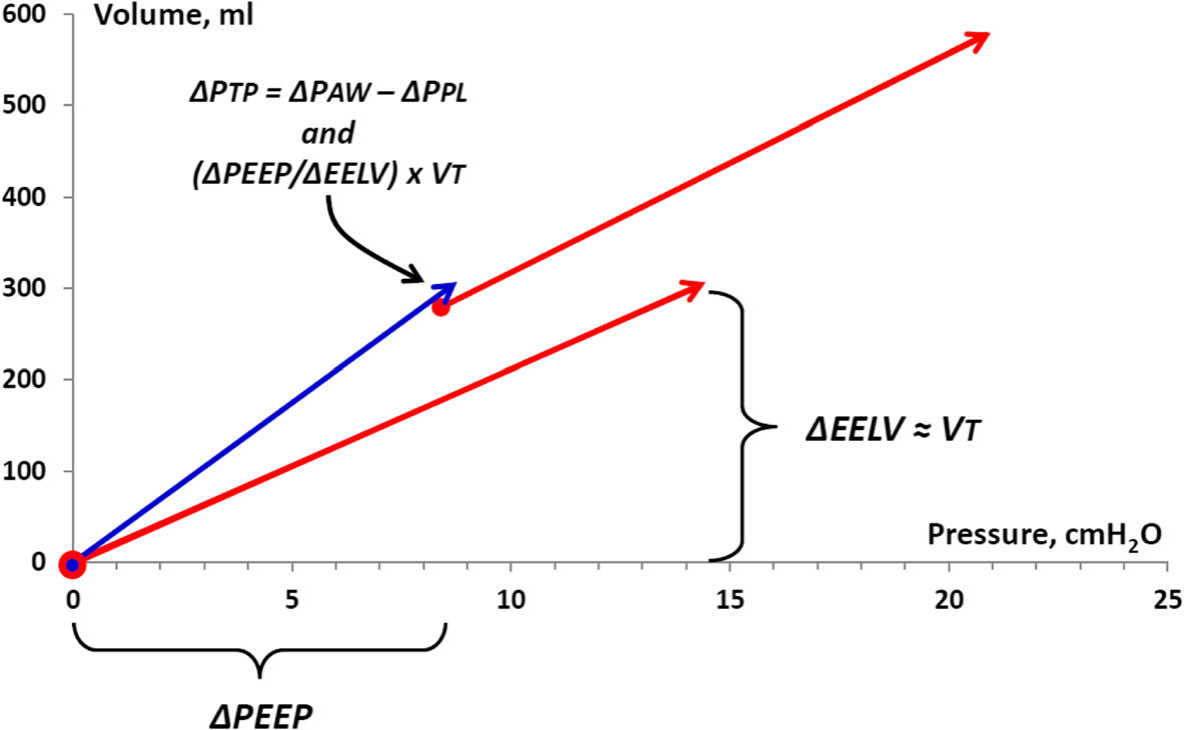
Tidal airway P/V curves of tidal volumes of 297 ml (*red arrows*) at ZEEP and 8.4 cmH_2_O of PEEP in the model with two test lungs (nominal lung compliance 38 ml/cmH_2_O). *Red circles*: end-expiratory airway P/V points, *arrows*: end-inspiratory airway P/V points. Transpulmonary P/V curve (*blue arrow*) of tidal volume of 297 ml. To determine the elastance of the lung, a volume change must be induced and the increase in transpulmonary pressure caused by the change in volume must be measured. A change in volume can be achieved by tidal inflation or by changing the end-expiratory airway pressure, PEEP inflation. In this case, changing PEEP by 8.4 cmH_2_O resulted in an increase in end-expiratory lung volume (281 ml) of the same amount as the tidal volume used (297 ml). In the experiment in the figure, the end-inspiratory transpulmonary pressure calculated conventionally as ΔPAW-ΔPPL is 14.1–5.3 = 8.8 cmH_2_O and calculated based on a PEEP step maneuver as (ΔPEEP/ΔEELV) × VT is (8/281) × 297 = 8.9 cmH_2_O

The consequence of the mean end-expiratory pleural pressure remaining at a baseline negative level when PEEP is increased is that the end-expiratory transpulmonary pressure increases as much as PEEP is increased. This is in accordance with findings in a porcine and in an ALI patient study, where the transpulmonary pressure variations of a tidal volume equal to the change in end-expiratory lung volume was closely related to the change in PEEP [8, 9]. As the transpulmonary pressure increases as much as the end-expiratory airway pressure changes, the static end-expiratory and end-inspiratory transpulmonary pressure at a certain lung volume level is independent of whether this volume is reached by tidal or PEEP inflation (Fig. 8). Consequently, end-expiratory and end-inspiratory transpulmonary pressure/volume points are aligned on a common, single transpulmonary (lung) P/V curve, as seen in the model, irrespective of whether one, two, or three test lungs are used, or the chest wall is normal or stiff (Figs. 2, 4, 5, 6, and 7).

An indication that the model is physiologically adequate would be if it could be shown, also in patients, that the increase in end-expiratory transpulmonary pressure following a PEEP increase is equal to the ΔPEEP. To confirm this, we calculated the increase in end-expiratory transpulmonary pressure of an end-expiratory lung volume increase following a PEEP increase as ΔEELV × EL from the reported mean values in 8 patients representative of patients with healthy lungs, 8 patients representative of moderate acute lung injury (ALI), and 8 patients representative of severe acute lung injury (ARDS) of a study by Pesenti and coworkers [39], and in 9 patients with pulmonary and 12 patients with extrapulmonary ARDS from a study by Gattinoni and coworkers [25]. In these representative “patients,” PEEP was increased from ZEEP to 5, 10, and 15 cmH_2_O in random order and the increase in lung volume was determined during a prolonged expiration to ZEEP from the different PEEP levels. In addition, the same calculation was performed in a group of 26 patients, representative of patients with mixed ALI, during incremental PEEP steps of 5 cmH_2_O, from 5 to 40 and back to 5 cmH_2_O [41]. In all three studies, lung elastance was determined as the tidal difference in airway pressure minus the tidal difference in esophageal pressure divided by the tidal volume, EL = (ΔPAW_LP_ − ΔPPL_LP_)/VT, avoiding uncertainties of the validity of absolute end-expiratory esophageal pressure in relation to absolute pleural pressure [6, 15, 42, 43, 44]. Twenty-eight PEEP steps in six mean “patients” representative of the most extreme lung conditions, from healthy lungs to the most severe pulmonary and extrapulmonary ARDS, were included and showed that the transpulmonary pressure increased 5.1 ± 0.7 cmH_2_O, when the PEEP was increased by 5 cmH_2_O and that end-expiratory pleural pressure, as a consequence did not increase, −0.1 ± 0.7 cmH_2_O. This is a strong indication that the model performs in accordance with human physiology as the increase in end-expiratory “pleural” pressure in the model also was negligible, 0.2 ± 0.1 cmH_2_O.

### Clinical implications

At end-expiration at any PEEP/EELV level below the resting volume of the chest wall, which is more than 3 l above FRC [30, 33], the spring-out force of the resilient rib cage makes the chest wall strive to a higher volume, while the end-expiratory airway pressure keeps the lung distended. The end-expiratory transpulmonary pressure and the end-expiratory pleural pressure at supine at FRC is zero at the most dorsal region of the *open* lung due to the gravitational pleural pressure gradient [11]. Thus, at end-expiratory pressure/volume equilibrium at increased PEEP, the end-expiratory transpulmonary pressure of most dorsal region of the *open* lung is equal to the end-expiratory airway pressure, as the chest wall is “lifted” from the lung, i.e., the chest wall does not squeeze or lean on the lung at end-expiration. It has been shown in a porcine study that the static transpulmonary pressure, PTPEE, is less damaging than the dynamic, transpulmonary driving pressure during tidal inspiration, ΔPTP [2, 45]. In humans, the key factor in ventilator-induced lung injury is the airway driving pressure [1], but in an accompanying editorial, it was pointed out that the culprit was not the total respiratory system (airway) but rather the transpulmonary driving pressure [3]. As the transpulmonary (lung) driving pressure can constitute anything between 50 % in mainly extrapulmonary ARDS and 90 % of the total respiratory system (airway) driving pressure in mainly direct, pulmonary ARDS, it is of great importance to be able to determine the lung driving pressure, both to know when there is a possibility to use higher airway driving pressure without harming the lung and when the tidal volume should be reduced to prevent harmful lung driving pressures. The present model shows that the ΔPTP can be calculated by a simple PEEP step procedure, where the change in end-expiratory lung volume is determined following a PEEP change. Then, lung elastance for the lung volume range between the two PEEP levels can be calculated as the change in PEEP divided by the change in end-expiratory lung volume, EL = ΔPEEP/ΔEELV. Tidal chest wall elastance can be determined as the difference between total respiratory system elastance (ΔPAW/VT) and lung elastance, chest wall elastance (ECW) = ETOT − ΔPEEP/ΔEELV. As tidal chest wall elastance is essentially constant when increasing PEEP, transpulmonary driving pressure at any PEEP level can be calculated as total respiratory system driving pressure minus tidal chest wall elastance times the tidal volume, ΔPAW − ECW × VT.

## Conclusions

*Tidal* chest wall elastance is related to the force needed to displace the abdominal content (weight) during *inspirations*. In contrast, end-expiratory chest wall elastance is related to the spring-out force of the rib cage, which maintains end-expiratory pleural pressure negative when increasing PEEP and is unaffected by conditions of the lung and the abdomen and remains mainly unchanged and close to zero in acute respiratory failure. Inspiratory and expiratory chest wall elastance are thus totally different entities, where inspiratory chest wall elastance is a measure of how much the chest wall and abdomen impedes lung inflation, while expiratory chest wall elastance indicates that the chest wall is off-loading the lung and consequently has no constraining effect on the lung at end-expiration.

As a consequence, we do not need to measure pleural or esophageal pressures to estimate lung elastance and changes in transpulmonary pressure.

## Abbreviations

ALI: Acute lung injury
ARDS: Severe acute lung injury
CW: Chest wall
ECW: Chest wall elastance
EE: End-expiratory
EELV: End-expiratory lung volume
EL: Lung elastance
ETOT: Total respiratory system elastance
FRC: Functional residual capacity
LP: Low PEEP
LV: Lung volume
P/V: Pressure/volume
PAW: Airway pressure
PEEP: Positive end-expiratory pressure
PPL: Pleural pressure
PPLEE: End-expiratory pleural pressure
PTP: Transpulmonary pressure
PTPEE: End-expiratory transpulmonary pressure = static transpulmonary pressure
TLC: Total lung capacity
VT: Tidal volume
ZEEP: Zero end-expiratory pressure
Δ…: Delta… = change in…
